# Designing text-messaging (SMS) in HIV programs: ethics-framed recommendations from the field

**DOI:** 10.11604/pamj.2015.21.201.6844

**Published:** 2015-07-16

**Authors:** Guillermo Martínez Pérez, Bella Hwang, Helen Bygrave, Emilie Venables

**Affiliations:** 1 Médicos Sin Fronteras/Doctors Without Borders Spain, Barcelona, Spain; 2 Doctors Without Borders South Africa, Johannesburg, South Africa; 3 Southern Africa Medical Unit, Doctors Without Borders South Africa, Cape Town, South Africa; 4 Medical Department, Médecins Sans Frontières, Operational Centre, Brussels, Belgium

**Keywords:** Ethics, mHealth, medical, HIV, Southern Africa, text messaging

## Abstract

Text messages (SMS) are being increasingly integrated into HIV programs across Southern Africa to improve patient adherence, linkage to care and provide psycho-social support. Careful attention needs to be paid to the design of SMS-based interventions for clients of HIV-care services to ensure that any potential harm, such as unwanted disclosure of HIV status, is minimized. In this article we propose a set of best practice recommendations to ensure that any SMS-based intervention considers ethical principles to safeguard safety, autonomy and confidentiality of its targeted HIV-positive beneficiaries. This analysis draws from our operational experience in Southern Africa in the design and conduct of mHealth interventions in the frame of HIV projects. The recommendations, framed in the context of the Belmont Report's three ethical pillars, may contribute to more safely operationalize any SMS service integrated into an HIV program if adopted by mHealth planners and implementers. We encourage actors to report on the ethical and methodological pathways followed when conducting SMS-based innovations to improve the wellbeing and quality provision of HIV-care for their targeted clients.

## Introduction

mHealth is a term that stands for the capitalization of telecommunications infrastructure and uptake of mobile information and communication technologies to support the provision of health services and achieve global, community and individual-level health priorities [1, 2]. mHealth has been identified as a potentially effective tool to improve the quality of HIV services [3]. The World Health Organization (WHO) has endorsed text messaging or short messages service (SMS) interventions for supporting individual-level adherence to antiretroviral therapy (ART) and for improving linkage of people diagnosed with HIV to HIV-care services [4]. In the hands of people living with HIV, SMS is a ubiquitous technology that holds promise to improve their health, psycho-social wellbeing and HIV-infection management. In this matter, SMS may be sent to remind HIV patients to adhere to their ART regime, attend their clinic and lab appointments, and to communicate medical examination results [5]. At community-level, SMS activities also support public health interventions, as messages can also be sent to the general population to promote the uptake of HIV counseling and testing (HCT) [5, 6] and encourage the use of condoms [7] and other HIV-prevention interventions such as male circumcision [8].

There is a paucity of documents explaining the ethical and theoretical models that inform the design of SMS-based interventions that target HIV-positive populations as mHealth program beneficiaries. Despite the developing evidence base of the effectiveness of mHealth as a patient support tool, it is still important to consider issues of confidentiality and privacy when designing an SMS program relating to a sensitive topic such as HIV. In relation to SMS-based behavioral interventions to improve adherence to ART, further research is required to explore whether patients will be able to respond to the messages and request additional healthcare support, and what the frequency, type and content of the messages should be in order to effectively influence patients’ health-seeking behavior [6, 9]. The potential harms of sending text messages must be considered in relation to other communication alternatives, especially in contexts such as sub-Saharan Africa, in which stigmatization towards people living with HIV is prevalent [10, 11].

### Aim and ethical framework

In this article we make recommendations for best-practice standard operating procedures to guide the design and implementation of SMS-based services to the beneficiaries of HIV-care and treatment. Our recommendations draw from field experiences of current mHealth studies and programs that are supported by the international humanitarian NGO Médecins Sans Frontiéres/Doctors Without Borders (MSF) in Malawi, Mozambique, South Africa, and Zimbabwe. All authors in this article are affiliated to MSF and have operational experience in the design and conduct of mHealth in the Southern African region. These recommendations are outlined in a three-pronged layout in accordance with the classic three ethical principles highlighted by the Belmont Report in 1979; beneficence, respect for persons, and justice [12]. In reporting the findings of our ethics-framed proposal, we use the word ‘client’ to refer to any target beneficiaries of a SMS-based service in the frame of a HIV program, be it people living with HIV, their caregivers or psychosocial or healthcare workers. Recent peer-reviewed literature on mHealth interventions targeting HIV-care beneficiaries is composed of program evaluations, operational research and experimental research [3, 5]. As research is conducted to assess the effectiveness and impact of mHealth, we also reviewed the Declaration of Helsinki to ensure that the recommendation made in this article do not conflict with the ethical principles set out to guide research [13]. It is our expectation that our recommendations could be adopted by mHealth planners and implementers to safely and ethically operationalize the development of any SMS-based service in the framework of an HIV-care and treatment program.

### Beneficence

**Figure 1:**
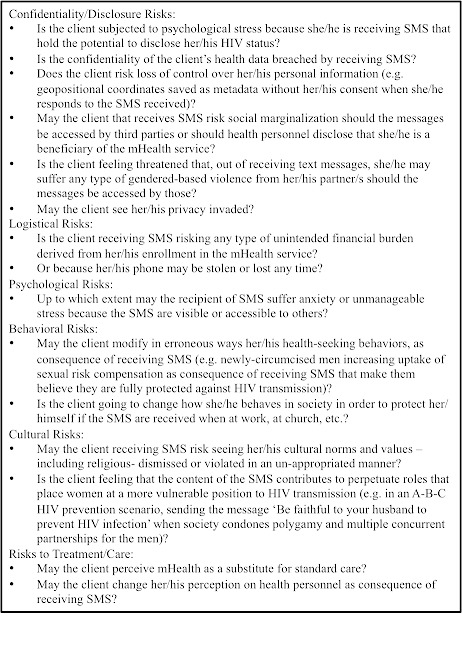
SMS potential adverse effects are of a different nature. Basic set of questions for planners to identify risks and harms

mHealth is deemed to do no harm to the individual beneficiary or to the community. Any stakeholder involved in mHealth service implementation has an ethical responsibility to maximize all possible benefits and minimize all possible harms. To apply this principle, a documented risk-benefit analysis document that informs the SMS intervention's design must be developed. Formative research may be conducted to accomplish this objective. Ethnographic and qualitative research methodologies can enhance understanding of behavioral, social and cultural dynamics of the clients as beneficiaries of any proposed mHealth service [14, 15]. Such a risk-benefit analysis can then assist with the implementation of mechanisms to prevent any identified harms. There are a range of harms that may be linked to receiving an SMS relating to HIV, including psychological, legal, social and cultural harms (Figure 1). Planners must escape from the monolithic analysis of risks based on the core assumption that sending an SMS relating to HIV leads to unwanted disclosure, and rather assemble an interdisciplinary team to explore potential harms from a more holistic perspective.

**Figure 2:**
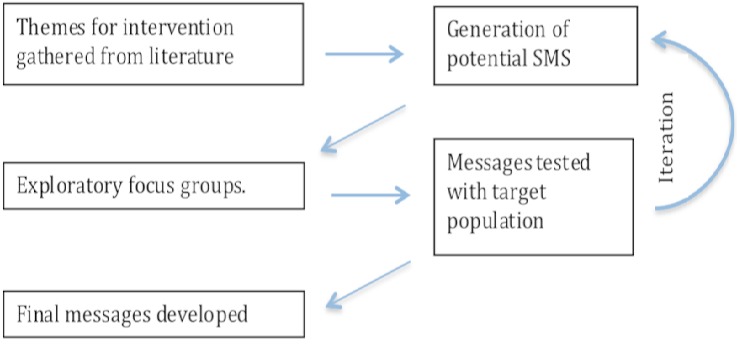
Framework for developing SMS messaging content

The design of SMS interventions, including the content of messages, the frequency in which they are sent and the language used, must be participative and iterative (Figure 2). Prior to implementation, we recommend consulting with a group of target clients and with a group of healthcare workers, as part of the foundational design process, to explore how to safely and appropriately design the SMS. It is useful to ask target clients to provide or narrate their life stories to improve the understanding of their personal circumstances and how receiving an SMS could be a beneficial or potentially harmful experience for them. Narratives obtained from HIV-care beneficiaries could assist to define a comprehensive catalogue of potential harms. Such information can be obtained via methods such as participant observation, face-to-face interviews, natural or focus group discussions or self-administered questionnaires and other participatory action research tools [16, 17]. For example, following this ethnographic approach to mHealth design and planning, MSF has involved HIV-positive individuals with the design of SMS messaging to communicate viral load results in Buhera district (Manicaland, Zimbabwe), and to develop encouraging reminders for ART adherence and linkage to care in uThungulu municipality (KwaZulu Natal, South Africa).

**Figure 3:**
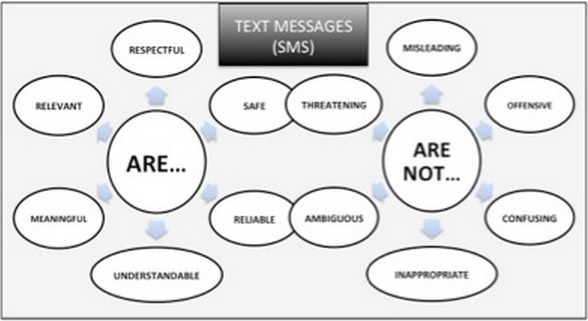
How Text Messages (SMS) should be (and should not be)

As well as asking potential clients for their opinions about the overall intervention, it is also useful to review draft messages with them to discuss the appropriateness of the content, tone and language used. It must be ensured that the final messages that the clients will receive are respectful, safe, reliable, understandable, meaningful and relevant (Figure 3) and in the case of HIV programs, that they will have a positive impact upon the health and well-being of the recipients. During this preliminary information gathering and analysis phase, it is also recommended to discuss safer alternatives to SMS to receive health information. Some clients may find it more convenient to receive an emoticon (a facial expression created by various combinations of keyboard characters), a phone call, paper-based pamphlet or recorded message, or not to receive anything and make use of a ‘Please Call Me’ or free callback service, or a toll-free or reverse-billed hotline instead. In certain instances, clients may feel that it is safer to receive coded or indirect messages as opposed to messages that include terminology directly relating to HIV. An iterative and participativory design process ensures that the decision on the omission of HIV-related terms, rather than being biased by the planner's assumption on the harms that SMS may involve, is ultimately based on the clients’ perceptions on how hazardous or intrusive receiving a decoded SMS is. Another issue worth exploring with clients is when best to send the SMS, as it may be safer and more appropriate to receive SMS at specific hours of the day. Others may think than receiving frequent SMS would put them at risk of unintentional HIV disclosure, with, for example, partners questioning who the messages are from. This might be the case, for instance, for newly diagnosed HIV-positive women who receive encouraging messages and whose mobile phones are accessible by their partners. These women may receive psycho-social support in the form of SMS supporting disclosure of their HIV status to their partners to encourage them to test, but they may risk gender-based violence if their HIV status is disclosed when receiving an SMS that overtly indicates the clients’ HIV status.

**Figure 4:**
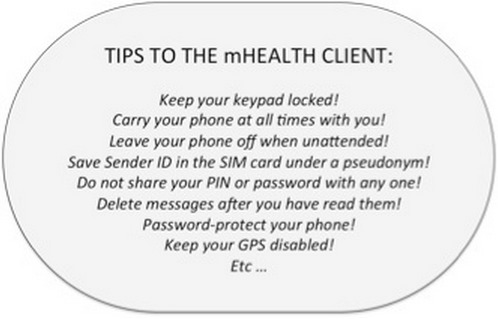
Basic tips to the clients to prevent potential harms

SMS-based interventions can include one-way or two-way communication [9]. In two-way interventions, clients may be asked to respond to the SMS with an answer relating to their current health status, or to confirm that they have received the information. This feedback may be useful for mHealth implementers to assess clients’ compliance with specific healthcare recommendations (e.g. report on ARV adherence, attendance to nurse consultations). The risks associated with clients sending a return SMS must also be assessed at this preliminary design stage. A local institution such as a healthcare facility might be requested to review the preliminary set of SMS agreed upon with the target clients. These messages can also be shared for official approval with the local government implementation partner to ensure the consistency of information and health promotion messages. To obtain final approval it is recommended to share the risk-benefit analysis and a thorough description of how ethical considerations will be addressed in agreement with the Belmont Report and the Declaration of Helsinki principles. During implementation, and as mHealth is still a relatively new field in HIV programming, an independent safety advisory board chaired by the local partners could be created to discuss unanticipated harms that arise from sending SMS, and to propose additional safeguards. Implementation can be piloted at small scale in a few sites, and the safety advisory board requested to review the clients’ response to the messages before the intervention is rolled out at scale. National data security and privacy regulations must be sought for, understood and analyzed in advanceas some countries have legislation that determines how SMS to health system beneficiaries must be delivered (or, if might be delivered at all). For example, in the United States all mHealth interventions must comply with HIPAA (Health Insurance Portability and Accountability Act), which is a U.S Federal regulation that provides standards for the provision of electronic healthcare [18]. Based on the pre-identified risks, the recommendations obtained through participative formative research, the restrictions and norms provided by local regulations and the directives given by government health institutions, a recommendation sheet should be prepared and handed to the target clients during the informed consent process. The recommendations must be intervention-tailored with the aim of minimizing all possible breaches in their safety, privacy and confidentiality (Figure 4).

### Respect for persons

In considering the ethical principle of ‘respect for persons’, the mHealth client must be treated as an autonomous being that makes free-of-coercion decisions about his/her own HIV-related healthcare goals and acts upon them. If the client is considered to be in a vulnerable position, he/she must be protected from coercion into enrolment in any mHealth service. To apply this principle, it should be a standard procedure to seek informed consent from all clients, who must voluntarily agree to receive SMS as part of the intervention. Numerous SMS interventions are being piloted in many government healthcare settings in Southern Africa and people are increasingly becoming familiar with SMS-based services for healthcare provision [19]. Hence, if deemed appropriate, and always ensuring that the clients receive all information beforehand, the consent to receiving SMS, rather than necessitating a separate informed consent document, can be integrated into existing clinic forms such as lab request forms or patients ART dispensing cards. For instance, in one of the abovementioned MSF-supported examples in Buhera (Zimbabwe), HIV-positive patients’ consent to receive a SMS reminder to return to the clinic to collect their last viral load test result, is collected on the ‘viral load request form’. Any healthcare worker (be it a counselor, nurse or clinician) that asks for consent must provide adequate information about the intervention, opportunity to consider all alternatives to receiving SMS (e.g. phone call, email, home tracing), ensure that the client comprehends all information and is given sufficient time to ponder all risks, and that all mechanisms are in place to request support in case any harm arises from the SMS. We recommend field-testing the consent process prior to implementing the SMS intervention as a means of ensuring that the information given to the clients is appropriate. The informed consent process can be piloted with a small sample of individuals sharing the socio-cultural characteristics of the target clients. If they are not fluent or literate in mobile technology, additional support materials such as videos, flip charts, pictorial menus or training tools might be designed during the field-testing, and then offered to future participants to enhance their understandability.

Sometimes, a third party may need to provide informed consent for a vulnerable person to receive messages. This may be the case for individuals such as adolescents under the age of legal consent for research, depending on specific country regulations [7, 20]. In the case of under 18s, youth assent should always be sought in addition to their guardian's consent. Local ethical advisory boards may be consulted if a waiver on seeking guardian consent is required. Illiterate individuals and the visually impaired who may not be able to read informed consent documents, could still provide oral consent. These populations can also benefit from SMS interventions as illiterate people could receive SMS that are converted into emoticons or pictorial messages, and visually impaired people may receive SMS that are converted into voice messages by mobile software [21]. For an informed consent process to be reliable, the added value of the SMS intervention and its potential disadvantages must be explained to the client. The clients should be made aware of why SMS is a more efficient option than other communication options (e.g. audio message, media message, phone call) or of any other care service option (e.g. facility-based face-to-face care). The clients should understand that mHealth is an appropriate solution for them, that they are not being deprived of other safer and more effective alternatives and that mHealth is not a substitute for any other conventional HIV-care service. If there is not yet an evidence base, the client needs to be reassured that the SMS-based service is being piloted and that it is being monitored for evidence of its effectiveness.

To ensure that no client is opted-in (registered) for the SMS program without fully understanding the potential harms and benefits, informed consent should only be undertaken by healthcare workers in a healthcare setting. As with any research study or intervention requiring informed consent, the autonomy of the clients to leave the SMS intervention at any moment without being requested to provide a reason and without penalized in any way, must also be emphasized. The person taking the consent must also provide instructions on how to opt-in at a later stage if the clients do not wish to be enrolled in the SMS intervention immediately, and how to opt-out (leave) at a later stage if they no longer wish to continue receiving SMS. For instance, in a MSF-supported intervention in Maputo (Mozambique), mothers that enroll in PMTCT B+ a vertical HIV transmission prevention program - are regularly given detailed instructions to opt-in and opt-out of a SMS messaging program that spans from mid-pregnancy until the point when a newborn receives a confirmatory HIV test. Instructions to opt-in and opt-out via mobile phone might be complicated for some end-users. In long-term mHealth scenarios (e.g. women receiving SMS during pregnancy and breastfeeding to prevent HIV transmission to the child) [9], to ensure that informed consent is regularly updated, the software that sends the SMS could be programmed to send a message with a permission prompt. However, we recommend that this way of renewing consent should not be used if the frequency or the content of the SMS is modified. In addition, if any client receives SMS against his/her will or should he/she express willingness to withdraw from the service, the text messages might be considered as spam, which is an illicit practice in many countries [22].

### Justice

Based on this ethical principle, the selection of mHealth beneficiaries must be justly distributed. The breach of the digital divide is progressively being reduced by increasing access to newer and cheaper technologies and through improved communication and information channels and networks. However, illiteracy, poverty and gender inequalities still prevent many people in sub-Saharan Africa from owning or using a mobile phone despite high coverage in the region [9, 23]. To apply this ethical principle it is recommended to monitor program enrollment. The recruiting agents must be regularly monitored in order to understand if barriers to access for potential clients are being created unnecessarily -within a particular health institution. Some vulnerable populations (e.g. men who have sex with men, commercial sex workers, and intersex and transgender people) might be prevented from enrolling in HIV healthcare services [10]. Vulnerable populations may be prevented from accessing care and treatment not only because of disease stigma and discrimination, but also because of other factors such as a lack of financial means to travel long distances to reach their closest healthcare facility. Issues around race, class, religious, political, or sexual orientation deterring eligible beneficiaries from opting into SMS services must be monitored, and mHealth planners should ensure that enrollment into a SMS intervention is also made accessible and affordable to vulnerable groups.

We don′t encourage the provision of incentives based on the number of clients enrolled so as to discourage the unfair enrollment of vulnerable clients who may experience harm as a result of receiving text-messages. MSF works in partnership with Southern African local government institutions to implement mHealth in public HIV-care facilities, thus no payment is provided to any healthcare worker in any of the mHealth scenarios described above. In compliance with this justice principle, and to ensure sustainability, SMS interventions aim to capitalize on existing staffing resources and be efficiently integrated within free government HIV-care and treatment services. Additionally, no incentives (financial or material) should be offered to potential clients to avoid vulnerable populations being coerced into enrolling for a service that may lead to harm. Socio-economic, cultural and gender disparities may hinder access to mHealth-based care for certain clients who, due to such disparities, do not have easy access to mobile hardware and communication services. Where gender inequality exists, many women in need of mHealth care may lack the means to access to cellphones, data and airtime [24]. In contributing to the breach of the digital divide and in promoting gender equality, registration into an mHealth service must be free of charge to participants. Either it should be reversed-billed to the implementing organization, or clients should only assume the costs of replying to messages and must be made aware of this during the informed consent process.

## Conclusion

In this article we have offered recommendations to guide the design and implementation of SMS interventions for those accessing HIV-related treatment and care. These recommendations draw from our experience in MSF-supported mHealth programs in Southern Africa. Our recommendations do not need to be adapted holistically, and program implementers may be interested in choosing the elements most relevant to their own projects and communities. However, we consider that if mHealth planners and implementers analyze their SMS-based interventions and frame them in a three-pronged structure according to the three ethical principles outlined in the Belmont Report, there is less chance that HIV-positive individuals will experience any potential harm that may be derived from receiving a SMS. We should also avoid the assumption that populations want decoded or indirect SMS, or that anyone receiving a medication reminder message or a viral load test result will automatically be stigmatized. We should systematically count on clients’ iterative participation in the design of any intervention they are to benefit from in order to maximize its potential impact. As this is a relatively new field of study, we also encourage scholars and implementers to thoroughly explain in their articles and progress reports the ethical and theoretical considerations in the design of their interventions. Scholars should put more emphasis into researching and documenting the frequency, type and content of the messages in order for mHealth interventions to meet their goal of influencing HIV-positive patients’ health-seeking behavior [9]. In reporting mHealth outcomes, we recommend that the relevant design benchmarks to be explained in detail should be: the step-by-step process of tailoring SMS to their target audience; the cognitive, learning and socio-behavioral theoretical models guiding the design process; which stakeholders are involved in the development of SMS interventions; the rationale behind or evidence supporting the assumption that SMS involves risks and should not include any HIV-related terms in the context of the message; and the process of seeking informed consent from the potential recipients of the SMS.

## References

[1] Mechael P, Batavia H, Kaonga N, Searle S, Kwan A, Goldberger A, Fu L, Ossman J (2010). Barriers and gaps affecting mHealth in low and middle countries: policy white paper; Colombia, USA. Centre for Global Health and Economic Development Earth Institute, Colombia University.

[2] World Health Organization (2011). mHealth: New horizons for health through mobile technologies: second global survey on eHealth.

[3] Catalani C, Philbrick W, Fraser H, Mechael P, Israelski DM (2013). mHealth for HIV treatment & prevention: a systematic review of the literature. The Open AIDS Journal..

[4] World Health Organization (2013). Consolidated guidelines on the use of antiretroviral drugs for treating and preventing HIV infection: recommendations for a public health approach.

[5] Free C, Phillips G, Galli L, Watson L, Feliz L, Edwards P, Patel V, Haines A (2013). The effectiveness of mobile-health technology-based health behavior change or disease management interventions for health care consumers: a systematic review. Plos Medicine..

[6] Mills EJ, Lester R, Thorlund K, Lorenzi M, Muldoon K, Kanters S, Linnemary S, Gross R, Calderon Y, Amico KR, Thirimurthy H, Pearson C, Remien RH, Mbuagbaw L, Thabane L, Chung MH, Wilson IB, Liu A, Uthman OA, Bangsberg D, Yaya S, Barnighausen T, Ford N, Nachega JB (2014). Interventions to promote adherence to antiretroviral therapy in Africa: a network meta-analysis. The Lancet..

[7] Cornelius JB, Jacek D, Boyer C, St Lawrence J, Lightfoot M, Moore M (2013). Text-messaging-enhanced HIV intervention for African American adolescents: a feasibility study. J Assoc Nurses AIDS Care..

[8] Odeny TA, Bailey RC, Bukusi EA, Simoni JM, Tapia KA, Yuhas K, Holmes KK, McClelland RS (2012). Text messaging to improve attendance at post-operative clinic visits after adult male circumcision for HIV prevention: a randomized controlled trial. PLoS One..

[9] Thirumurthy H, Lester RT (2012). M-health for health behaviour change in resource-limited settings: applications to HIV care and beyond. Bulletin of the World Health Organization..

[10] Ntoh Yuh J, Ellwanger K, Potts L, Ssenyonga J (2014). Stigma among HIV/AIDS patients in Africa: a critical review. Social and Behavioral Sciences..

[11] UNAIDS, Global Report (2013). UNAIDS report on the global AIDS epidemic 2013.

[12] United States Department of Health and Human Services (UDDHHS) (1979). The Belmont Report: ethical principles and guidelines for the protection of human subjects of research. The National Commission for the Protection of Human Subjects of Biomedical and Behavioral Research.

[13] World Medical Association (2008). Declaration of Helsinki: ethical principles for medical research involving human subjects.

[14] Martínez Pérez G, Massaguer Pla D (2012). Remote usability study on mHealth app VirTelMed in a South African setting. International Journal of Computer Applications..

[15] Mechael PN (2009). MoTECH mHealth Ethnography Report..

[16] Collumbien M, Busza J, Cleland J, Campbell O (2012). Social science methods for research on sexual and reproductive health.

[17] Kielmann K, Cataldo F, Seeley J (2012). Introduction to qualitative research methodology: a training manual; United Kingdom. Department for International Development (DfID).

[18] Group Speciale Mobile Association (2014). mHealth Tracker.

[19] Luxton DD, Kayl RA, Mishkind MC (2010). Health data security: the need for HIPAA-compliant standardization. Telemed J E Health..

[20] Cornelius JB, Cato M, St Lawrence J, Boyer CB, Lightfoot M (2011). Development and pretesting multimedia HIV-prevention text messages for mobile cell phone delivery. J Assoc Nurses AIDS Care..

[21] Baravkar SR, Borde MR, Nivangune MK (2013). Android text messaging application for visually impaired people. IRACST Engineering Science and Technology: an International Journal..

[22] Wang C, Zhang Y, Chen X, Liu Z, Shi L, Chen G, Qiu F, Ying C, Lu W (2010). A behavior-based SMS antispam system. IBM Journal of Research and Development..

[23] Ericsson (2013). Bridging the digital divide. How mobile phones are playing a key role in connecting people in Sub-Saharan Africa.

[24] Deshmukh M, Mechael P (2013). Addressing gender and women's empowerment in mHealth for MNCH.

